# Special Issue: Wine and Vine Components and Health

**DOI:** 10.3390/diseases7010030

**Published:** 2019-03-19

**Authors:** Norbert Latruffe, Jean-Pierre Rifler

**Affiliations:** 1 Université de Bourgogne, 21000 Dijon, France; 2 Haute Côte d’Or Hospital Center, F-21350 Montbard, France

There is much literature on the topic of wine and health dating back to the days of Hippocrates, and it is believed that there are unlimited varieties of wine, allowing the association of senses, nutrition, and hedonism. The history of vine and wine has lasted for at least 7000 years (Latruffe, 2018 [1]). *Vitis* is an adaptable plant, thanks to a large variety of strains; wine is an alchemy with unique properties, a rich and original composition in terms of polyphenols and well-known antioxidants (Figure 1, see below). This explains why wine and health are closely linked to nutrition (Latruffe, 2017 [2]).

In terms of biochemical mechanisms, vines like other plants produce numerous non-energy compounds, called secondary metabolites (e.g., flavonoids, polyphenols), in order to adapt their defenses against an often unfavorable environment (biotic and non-biotic stresses). Interestingly, in humans and in the animal kingdom these microconstituents provide similar valuable bioactive properties for essential cell and physiological function (signaling, gene regulation, prevention of acquired or infectious disease, etc.). These compounds have been selected through evolution and are generally preserved in all living beings. For instance, resveratrol that plays an essential role in vine plants as elicitor of natural defenses has been shown to be a protector of health in humans. It can delay, or even block, the appearance of predominant diseases such as atherosclerosis by protecting low-density lipoproteins from oxidation, but also diabetes and cancer.

The grape, fresh or dried, is a fruit widely consumed by large human populations, as well as its by-products such as grape juice and wine. Some even use vine leaf extracts and vine shoots. Grapes contain vast and highly varied quantities of polyphenols as a protective micronutrient. Wine provides unique polyphenols—for instance, resveratrol, procyanidines, and monophenols such as hydroxytyrosol and tyrosol. Research supports the idea that wine, which is a natural biological product, if consumed regularly but without excess, possesses preventive properties, not only having its well-known properties against vascular diseases (illustrated by the so-called French paradox) but also possibly preventing infections, decreasing inflammation, and delaying neurodegenerative diseases. The question with respect to cancer is still open.

Despite the huge amount of data on this topic, gray areas still remain and knowledge is incomplete. That is why the objective of this issue is to present a better view of wine, especially through policy makers, the medical world, and the vectors of image in order to explain the justification and the philosophy of wine with respect to ethics and public health.

This Special Issue of the journal *Diseases* focuses on wine and vine components and health and includes the effects of wine on human physiology (cardiovascular diseases, aged-linked disorders, etc.); the effects of polyphenols as wine antioxidants and as signaling molecules; and, from a humanity point of view, the tasting properties of wine.

We edited four primary articles and five reviews providing new data and new concepts related to the following keywords: antioxidant capacity, wine, vine, and grape components, including ethanol and polyphenols such as resveratrol, and flavonoids; their metabolism and their effect on pathologies such as aging, longevity, vascular diseases, diabetes, cancer, inflammation, allergies, neurodegeneration, among others. The paper entitled “Is a Meal without Wine Good for Health?” by Jean-Pierre Rifler [3] has been selected as the issue cover.

The new findings from original articles are as follows.

Concerning innovative technology, a paper reports on an Electrochemical Method for Evaluating Antioxidant Capacity of Wines, called PAOT (”Pouvoir Anti-oxydant Total”). Using this method, the authors found that the total antioxidant activity was almost seven-fold higher in red wines when compared to rosé and white wines from the commercial market. Winemakers can use PAOT to evaluate the antioxidant activity of wine during the winemaking process (Pincemail et al., [4]). 

A case control study was carried out by Boronat et al. [5] on wine and olive oil phenolic compounds and metabolism in humans. They studied the metabolism of resveratrol (from red wine), and of tyrosol and of hydroxytyrosol (from red wine and from extra virgin olive oil) and found an increase in urinary tyrosol and hydroxytyrosol from a combination of red wine and extra virgin olive oil intake, whereas resveratrol remained identical as red wine intake only.

With the aim of slowing neurodegeneration associated with aging, especially Alzheimer’s disease and Parkinson’s disease, the effects of resveratrol and other Mediterranean diet-associated polyphenols have been studied with respect to neuronal differentiation (Namsi et al., [6]). Interestingly, they found that resveratrol and apigenin can induce cultured cell neuronal differentiation.

A preclinical study on spontaneously hypertensive rats (SHR) was performed to analyze the remaining potential of grape by-products from various red wine cultivars (Rasines-Perea et al.; [7]). Extracts used from grenache, syrah, and alicante cultivars presented a ”rebound effect” on systolic blood pressure, whereas the other extracts (carignan, mourvedre, etc.) showed no significant changes.

Review papers presented current knowledge on different subjects featured in the Special Issue.

Tanaka et al. [8], reported on the potential beneficial effects of wine flavonoids on allergic disease models, but the evidence in humans is limited to allergic rhinitis and respiratory allergy.

Vervandier-Fasseur’s group [9] selected the synthesis of innovative *trans*-resveratrol derivative procedures, in order to increase its solubility in water and pharmacological activities toward cell targets.

The potential effects of polyphenol extracts from red wine and grapevine on cancers have been summarized by Amor et al. [10]. They discuss how the polyphenolic composition of red wine may influence its chemopreventive properties.

Pavlidou et al. [11] compared wine to an aspiring agent in promoting longevity and preventing chronic diseases. They especially highlight the beneficial role of red wine against oxidative stress and in favor of desirable gut bacteria, so-called microbiota, where some promising studies are pending.

After having recalled that wine is the elixir that, by design and over millennia, has acted as a pharmacopeia that has enabled people to heal and prosper on the planet, Rifler [3] pointed out the characteristics of wine drinking linked to religion, culture, civilization, and the manner of eating (insisting on the Cretan and Okinawa diets). He finishes with the following message:”Moderate drinking gives a protection for diseases and a longevity potential. In conclusion, let us drink fewer, but drink better, to live older.” 

This Special Issue of *Diseases* focusing on the effects that wine and vine components have on health allows us to publish new findings on antioxidant capacity measurement using innovative technology, on the metabolism of polyphenols with respect to humans, on the induction of neuron differentiation in cell models by resveratrol, and on the regulatory effect of hypertension in animals by some wine by-products. On the other hand, reviews make statements on wine polyphenols in connection with allergy/inflammation, with cancer, with intestine microflora, and with diet. Finally, we learn about perspectives opened by new resveratrol derivatives to fight low bio-availability of the parent molecule.

## Figures and Tables

**Figure 1:**
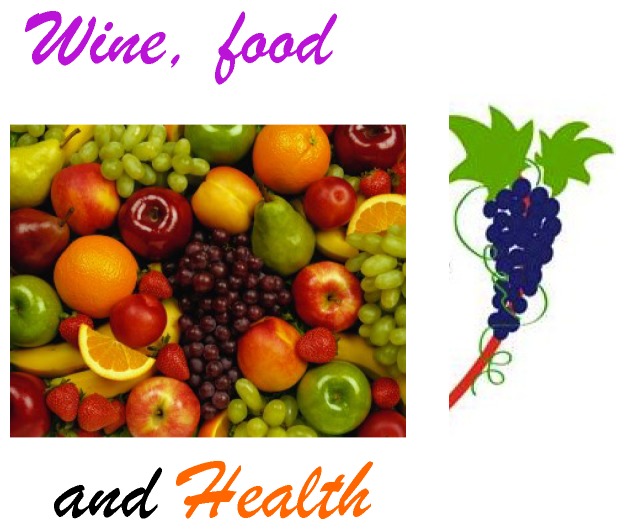
Figure of the authors.
